# Low Blood ALT Activity and High FRAIL Questionnaire Scores Correlate with Increased Mortality and with Each Other. A Prospective Study in the Internal Medicine Department

**DOI:** 10.3390/jcm7110386

**Published:** 2018-10-25

**Authors:** Gringauz Irina, Cohen Refaela, Brom Adi, Davidi Avia, Hofstetter Liron, Avaki Chen, Segal Gad

**Affiliations:** 1 Department of Internal Medicine and Geriatrics “C”, The Chaim Sheba Medical Center, Tel-Hashomer, Affiliated to the Sackler Faculty of Medicine, Tel-Aviv University, Ramat-Gan 5265601, Israel; irina.gringauz@sheba.health.gov.il; 2 Department of Internal Medicine “T”, The Chaim Sheba Medical Center, Tel-Hashomer, Affiliated to the Sackler Faculty of Medicine, Tel-Aviv University, Ramat-Gan 5265601, Israel; refaela.cohen@sheba.health.gov.il (C.R.); adi.brom@sheba.health.gov.il (B.A.); davidai@sheba.health.gov.il (D.A.); liron.Hofstetter@sheba.health.gov.il (H.L.); chen.avaki@sheba.health.gov.il (A.C.)

**Keywords:** frailty, sarcopenia, survival, internal medicine, FRAIL questionnaire, ALT

## Abstract

Background: Low blood ALT, Alanine aminotransferase activity and high FRAIL (Fatigue, Resistance, Ambulation, Illnesses and Loss of Weight) questionnaire scores were previously shown to be associated with frailty and increased risk of mortality. We aimed to correlate these tools with mortality and each other in patients hospitalized in an internal medicine department. Methods: This is a prospective study in a large tertiary hospital. We assessed the predictive value for clinical outcomes of both low ALT blood activity and the pre-frail and frail categories of the “FRAIL” questionnaire. Results: During a 15 months study, 179 consecutive patients were recruited, of whom 20 died. When all study participants were divided to three groups according to admission ALT levels (below 10 IU/L, 11 to 19 IU/L and above 20 IU/L) we found a statistically significant difference in the rate of mortality: 4 patients died within the group of ALT < 10 IU/L, 14 patients died in the group of 10 IU/L < ALT < 19 IU/L and in the group of patients with ALT > 20 IU/L, only 2 patients died (*p* = 0.042). A higher score on the FRAIL questionnaire was associated, with statistical significance, with higher risk of mortality (*p* = 0.029). There was a significant correlation (*p* = 0.038) between blood ALT activity and the pre-frailty and frailty classifications by the FRAIL Questionnaire. Conclusions: Both the FRAIL questionnaire and blood ALT activity are simple and practical tools for frailty assessment and risk stratification of patients hospitalized in the internal medicine department. Both tool’s results also correlate with each other.

## 1. Introduction

For the elderly population, the assessment and definition of frailty are still under development and are debated in the literature [1,2]. Regarding the non-elderly, middle-aged population, the terms frailty, sarcopenia and their association with survival in the literature are indeed scarce. Some authors searched for the impact of frailty in sub-populations included in the realm of the general internal medicine: Uchmanowicz and co. investigated the frailty syndrome and its association and impact on arterial hypertension [3]; Abdel-Kader and co. investigated the frailty following acute kidney injury [4], Abel and Klepin proposed frailty assessment to replace the patients’ age as a predictor of responses to therapy in various hematologic malignancies [5]. Nevertheless, none has questioned the value of frailty and sarcopenia assessment within the internal medicine departments themselves. 

Indeed, the patient population in the internal medicine department is heterogeneous and challenging. Even more challenging are ways to attain a holistic tool for prognostication in this population. In this study, we tried to establish means, which could be available and correlate with our patients’ frailty status and general prognosis. This study was intended to serve as the basis for a larger prospective study, which would not only be larger but would also relate to specific morbidities that are common in the internal medicine daily practice (congestive heart failure, chronic obstructive pulmonary disease etc.). 

The two frailty assessment tools used in this study are ALT, Alanine aminotransferase blood activity measurement and the FRAIL (Fatigue, Resistance, Ambulation, Illnesses and Loss of Weight) questionnaire. For the ALT measurement for assessment of sarcopenia and frailty, our group has published several studies, establishing association between low-ALT values, sarcopenia, frailty and increased risk of all-cause-mortality. The current knowledge regarding ALT can be summarized that this is a good biomarker for sarcopenia and frailty (in most publications sarcopenia is correlated when ALT is of low-normal values, in the range of 10 to 17 IU) [6,7,8,9,10]. The main advantages of ALT measurement are its high availability, low cost and lack of side effects and the fact that it reflects a whole-body phenomenon, rather than examining one or several sites in the body (as opposed to imaging studies and anthropometric measurements. Regarding the FRAIL questionnaire, it includes 5 components: Strength, assistance in walking, rising from a chair, climbing stairs, and past falls. The FRAIL scores represent frail (3–5), pre-frail (1–2) and robust (0) health statuses. The FRAIL questionnaire was validated in a group of African Americans, age 49 to 65 years [11]. Morley and co. did a cross-validation and found that the FRAIL scale correlated significantly with IADL (Instrumental Activities of Daily Living) difficulties, handgrip strength and one-leg stand among participants with who had no baseline ADL (Activities of Daily Living) difficulties. Such a correlation was also demonstrated for subjects with no baseline ADL problems. The results of this longitudinal study showed that the “pre-frail” definition by FRAIL at baseline was associated, with statistical significance, with future ADL difficulties, worse one-leg standing, and mortality. Scoring as “frail” in the FRAIL at baseline was associated with future ADL difficulties, IADL difficulties, and increased risk of mortality. 

No previous studies evaluated ALT and/or the FRAIL questionnaire in the population of hospitalized patients. Moreover, no previous study questioned the possible association of these tests with each other and with clinical correlates. 

## 2. Methods

This was a prospective study in consecutive patients hospitalized to an internal medicine department of a large, tertiary hospital. Study participants were recruited after the approval of an institutional ethics committee. Inclusion criteria for participation included: (1) Over 18 years of age; (2) ALT levels upon admission within normal limits (lower than 40 IU/L); (3) later to be divided into 3 groups according to previously published cut-offs. Ability and will to answer the FRAIL questionnaire. Exclusion criteria were: (1) Dementia and other situations associated with cognitive decline (such as use of sedatives); (2) Follow-up data becoming unavailable during the study follow-up period. Demographic data and co-morbidities were documented in accordance with the computerized patients’ records during hospitalization. Blood ALT levels were documented from the routine chemistry analysis (using a standard Beckman Diagnostics Coulter^®^, Brea, CA, USA). taken from each patient upon admission (during the first 24 h of hospitalization). ALT blood levels were calculated according to their catalytic activity (measured by IU, International Units, in standard chemistry tubes supplemented with activated pyridoxal phosphate P-5-P, acting as a co-factor for ALT). FRAIL questionnaires were filled by patients with the assistant of a stuff member when needed. Questionnaires were also introduced to patients during their first 24 h after admission. 

### Statistical Methods

Categorical variables were expressed as frequency and percentages. Distribution of continuous variables was assessed using histogram and Q-Q (Quantile-Quantile) plots. Normally distributed continuous variables were described using mean and standard deviation (±SD), and non-normally distributed continuous variables were expressed using median and interquartile range (IQR). Chi-square test or Fisher’s exact test were used to evaluate association between categorical variables. Correlation between continuous variables was evaluated using Spearman’s correlation coefficient. A two-tailed *p* < 0.05 was considered statistically significant. Multivariate analysis included variables found to have significant association with end-points on univariate analysis. All analyses were performed with IBM SPSS Statistics for Windows, Version 22.0 (IBM Corp., Armonk, NY, USA).

## 3. Results

### 3.1. Patients’ Characteristics

During a 15 month period, we recruited 179 consecutive patients that fulfilled our study’s inclusion criteria and signed an informed consent form. After a maximal follow-up period of 456 days, 20 patients died (most of them died after hospital discharge, and the end-point was, therefore, referred to as “all-cause mortality”). Main patients’ characteristics are described at Table 1, presenting various background patients’ data that are potentially associated with their prognosis: Epidemiological data included age, gender and Norton score, anthropometric data included weight and BMI (Body Mass Index), relevant background morbidities included chronic diseases like heart failure, diabetes and malignancy, relevant laboratory values (commonly acting as confounders) were also included: Kidney function tests, albumin and hemoglobin concentration. 

### 3.2. ALT Levels and Clinical Correlates

**Figure 1:**
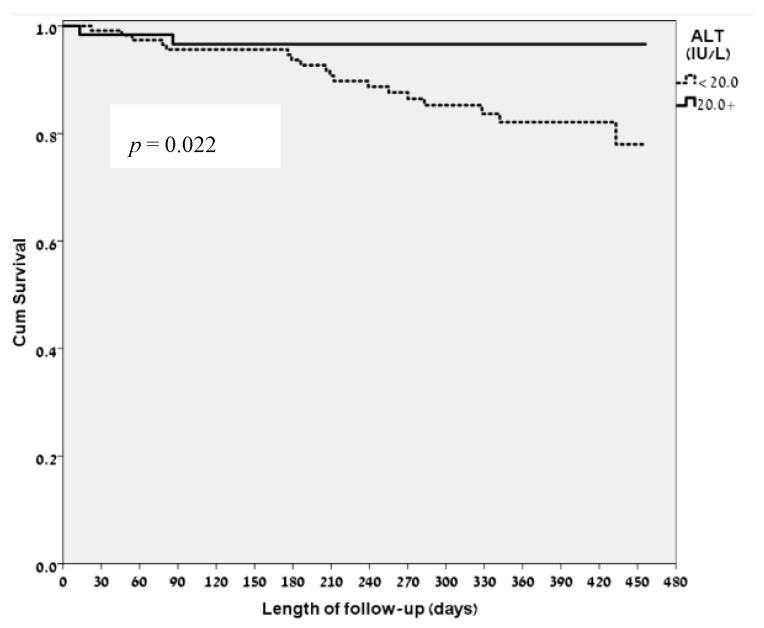
Kaplan-Meier survival curve according to ALT, Alanine Aminotransferase levels on admission: below and over 20 IU/L.

**Figure 2:**
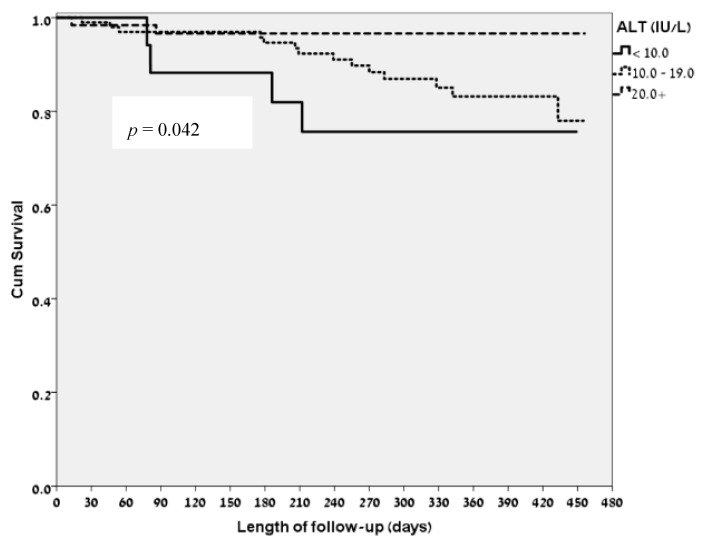
Kaplan-Meier survival curve according to ALT groups on admission: below 10 IU/L, between 10 to 19 IU/L and over 20 IU/L.

The mean ALT level in our study population was 17.83 IU/L. Amongst the patients who died during the follow-up period, the mean ± SD ALT levels were 14.1 ± 5.6 IU/L while amongst surviving patients the mean ± SD ALT levels were 18.28 ± 7.88 IU/L, (*p* = 0.022). Amongst the 20 patients who died during the follow-up period there were only 2 patients whose ALT level on admission was greater than 20 IU/L. Figure 1 show the Kaplan Meier survival curve according to ALT levels on admission. When all study participants were divided to three groups according to their ALT levels on admission; (below 10 IU/L; (*n* = 18, 11 to 19 IU/L; (*n* = 99) and above 20 IU/L; (*n* = 62); the lowest cut-off value was pointed according to the study by Gringauz, Weismann and Justo et al. [9]), we found that there was a statistically significant difference in the rate of mortality: 4 patients died within the group pf ALT < 10 IU/L, 14 patients died in the group of 10 IU/L < ALT < 19 IU/L and in the group of patients with ALT > 20 IU/L only 2 patients died (Figure 2; *p* = 0.042). 

Within the range of Low and normal ALT values, we found that higher ALT levels on admission were associated with decreased risk of mortality: In a univariate analysis, patients with ALT > 10 IU/L had a 7.5% lower risk of death during a maximal follow-up period of 456 days (HR = 0.925, 95%CI 0.86–0.99; *p* = 0.034). In a multivariate analysis, taking into account both low ALT, age and gender, only low ALT was found to be associated with an increased risk of mortality (*p* = 0.029).

### 3.3. FRAIL Questionnaire Classifications and Clinical Correlates

**Figure 3:**
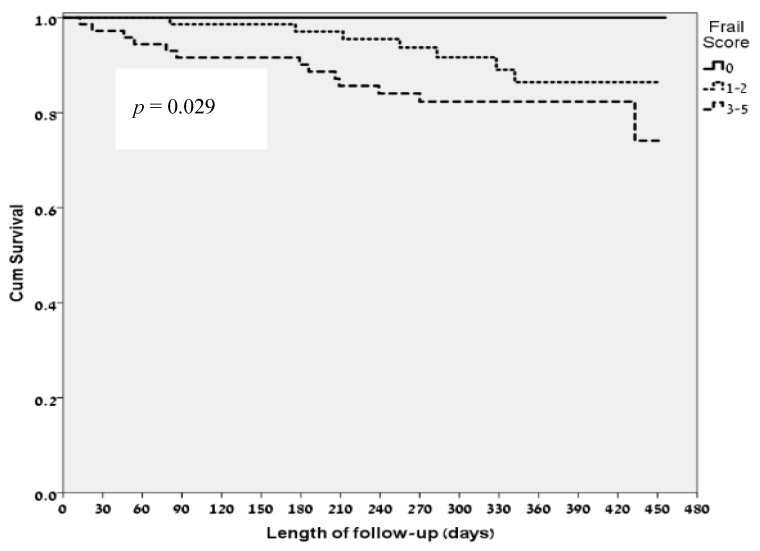
Kaplan-Meier survival curve according to FRAIL Questionnaire.

Amongst patients who were classified as “robust” (score 0, *n* = 30) by the FRAIL questionnaire there were no mortality events during the study period. Amongst patients who were classified as “pre-frail” (scores 1–2, *n* = 77) by the FRAIL questionnaire, there were 7 deaths during the study period and amongst patients who were classified as “frail” (scores 3–5, *n* = 72) by the FRAIL questionnaire, there were 13 deaths during the study period. Overall, a higher score on the FRAIL questionnaire was associated, with statistical significance, with a higher risk of mortality (Figure 3; *p* = 0.029).

### 3.4. Correlation of Low ALT Levels and the FRAIL Questionnaire Ggroups

**Figure 4:**
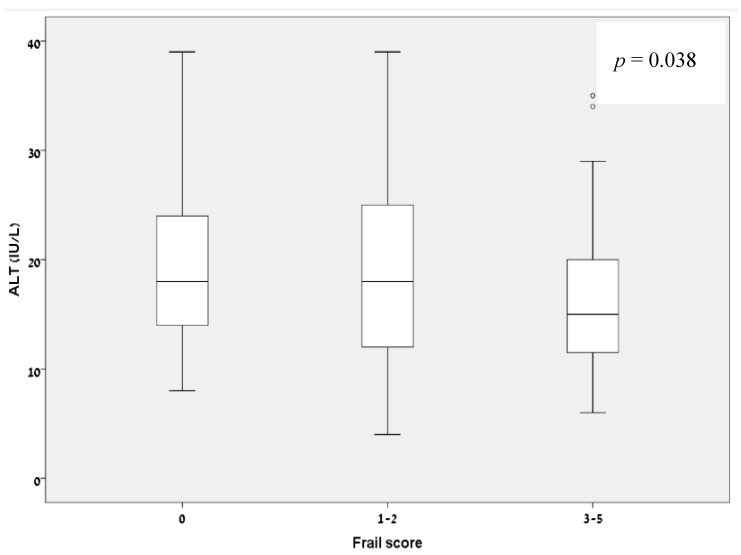
Low ALT values correlate with FRAIL Questionnaire groups.

There was a statistically significant correlation between blood ALT levels and pre-frailty and frailty classifications as reflected by the different groups of the FRAIL Questionnaire (Figure 4; *p* = 0.038): amongst patients whose FRAIL score was 0 (robust) the median (IQR) ALT level was 18 IU/L (13.75–24.25). Amongst the pre-frail participants (FRAIL scores 1 to 2) the median (IQR) ALT levels were 18 UI/L (12–25). For patients who were defined as frail by the FRAIL groups 3 to 5, the median (IQR) ALT level was 15 IU/L (11.25–20). 

## 4. Discussion

Although frailty has been provided with many definitions, the one most suitable was set by Abellan and co. in their task force (on behalf of the International Academy of Nutrition and Aging) for frailty assessment in the clinical practice: They termed frailty as a pre-disability phase, predictive of future disability, morbidity, institutionalization and death [12,13]. That same task force used the FRAIL questionnaire as a well validated frailty model.

### 4.1. The FRAIL Questionnaire, Validation and Applications

Morley and co. have conducted a prospective validation of the FRAIL questionnaire (FQ) in a population of middle-aged (49 to 65 years) African Americans. They found that being pre-frail, according to the FQ at baseline, predicted future ADL difficulties and long-term mortality [8]. Moreover, being frail, according to the FQ, predicted both ADL and IADL difficulties and mortality in the future. The difference between the pre-frail and the frail categories was relevant only when the study population was divided to ADL dependent and ADL non-dependent participants at baseline: Being pre-frail predicted IADL dependence only in the dependence-excluded group while being frail added a worse SPPB (Short Physical Performance Battery protocol) in the dependence-excluded group. The FQ was also used by Hyde, Flicker and Almeida et al. they found out that lower free testosterone levels, assessed in 3616 non-disabled men aged 70 to 88 years, was associated with frailty as appreciated by the FQ scores [14]. The current study results support the hypothesis that FQ could serve as a valid tool for frailty assessment in the vast population of patients hospitalized in internal wards. 

### 4.2. Low ALT, Sarcopenia, Frailty and Survival

Alanine aminotransferase activity in the blood is commonly measured as a marker for cellular, liver injury, whereby its levels are elevated. Less is known regarding the circumstances in which low ALT levels are measured in the blood. Low ALT levels could be assigned to several possible explanations: 1. Low vitamin B6 and its metabolite, Pyrydoxal-5-phosphate (P5P) activity: Since P5P serves as a co-factor for ALT actions, low P5P levels could account for low ALT activity. It was shown that the potential impact of adding P5P or eliminating it from ALT test tubes could account for 10% to 20% variability of ALT test results [15].

In a 4.9 years follow-up of 1673 community dwelling elderly men, Le Couteur and co. showed that low Alanine amino transferase (ALT) could be a surrogate marker for sarcopenia, frailty and shortened survival [16]. Low-normal ALT, when inspected amongst middle-aged people whose ALT was within the normal range (up to 40 IU/L) was found to be associated with increased, all-cause mortality, even after a multivariate model corrected potential bias of age, gender, renal function and blood albumin level. This was shown by Ramaty, Maor and Peltz-Sinvani et al. in a retrospective analysis of 23,506 people over a period of 8.5 years [6]. Ramaty and co. also showed that low ALT activity in the blood is a common finding amongst hospitalized patients [7]. In another long-term follow up cohort (6575 participants of the BIP (Bezafibrate Infarction Prevention) study, followed for more than 22 years), Peltz-sinvani, Klempfner and Ramaty et al. also showed that low ALT was associated with increased risk of mortality [8]. 

Gringauz, Weismann and Justo et al. [15] showed that ALT blood levels greater than 10 IL/U amongst elderlies who suffered hip fractures, before rehabilitation, were associated with a better rehabilitation outcomes: Logistic regression analysis adjusted for age and gender showed that patients with ALT over 10 IU/L were more likely to have higher total FIM (Functional Independence Measure) scores (>50), cognitive FIM scores (>16), and FIM efficiency (>0.228) upon rehabilitation discharge (Odds Ratio = 1.56–1.78) following hip fracture surgery.

All the above are evidence for low ALT being a biomarker for sarcopenia and frailty. Still, the standards for “low ALT” are still missing. For this reason, we detailed our results regarding two different sets of ALT measurements: below and above 20 IU (at the point of 50% of highest normal values) and within this range: Round the 25% point of highest normal value (at 10 IU).

The current study is the first ever showing the value of applying, in a prospective manner, both the FRAIL questionnaire and ALT tests in a population of hospitalized patients, for the purpose of frailty assessment and prognostication. Moreover, this is the first report of the statistically significant association of low ALT values and the pre-frail and frail domains of the FRAIL questionnaire results.

### 4.3. Study Limitations

This was a prospective, single-center, study in a small number of patients. The results should be reinforced in larger scale, multi-center studies. We also encourage appreciation of assessment tools other that those that we used (e.g., anthropometric measurement tools).

## 5. Conclusions

The results of this study show that application of simple and available tools for sarcopenia and frailty assessment of patients admitted to internal medicine departments is both practical and worthwhile. The fact that these patients’ characteristics correlate with their prognosis, emphasizes that we are dealing with true personalization of internal medicine. In contrast to higher precision (namely, better targeting of medications to pathologies), sarcopenia and frailty assessment are tools for better personalization of therapy. The authors assume that application of our findings, could better adjust treatment protocols for patients hospitalized in internal medicine departments. It should be stated that this is our point-of-view and further, prospective studies are needed in order to further establish our clinical approach. 

## Figures and Tables

**Table 1:** Patients’ Characteristics.

Patients’ Demographics	
Age (years, Median (IQR))	72 (65–79)
Female gender (%)	46.4
Norton score (Median (IQR))	19 (18–20)
Body weight (Kg, Median (IQR))	75 (65–84.5)
BMI (Median (IQR))	26.7 (23.6–29.8)
**Comorbidities (%)**	
Cardiovascular disease	43.2
Diabetes mellitus	33.1
Respiratory disease	12.7
Infectious disease	12.2
Background malignancy	12.2
**Laboratory Data**	
Creatinine (mg/dL, (Median (IQR))	0.86 (0.68–1.15)
Urea (mg/dL, (Median (IQR))	38 (29–52)
Albumin (mg/dL, (Median (IQR))	3.7 (3.3–4.0)
Hemoglobin (gr/dL, (mean ± SD)	11.8 ± 2.2
